# Androgen-Induced Cardiovascular Risk in Polycystic Ovary Syndrome: The Role of T Lymphocytes

**DOI:** 10.3390/life13041010

**Published:** 2023-04-14

**Authors:** Mohadetheh Moulana

**Affiliations:** Department of Psychiatry and Human Behavior, Women’s Health Research Center, University of Mississippi Medical Center, 2500 North State Street, Jackson, MS 39216, USA; mmoulana@umc.edu

**Keywords:** polycystic ovary syndrome, hyperandrogenemia, inflammation, hypertension, Th17 cells, CD4^+^CD28^null^ T cells, cardiovascular risk factor

## Abstract

An estimated 15–20% of reproductive-age women are affected by polycystic ovary syndrome (PCOS). PCOS is associated with substantial metabolic and cardiovascular long-term consequences. In young women with PCOS, several cardiovascular risk factors may be found, including chronic inflammation, high blood pressure, and elevated leukocytes. These women are at an increased risk of cardiovascular diseases (CVD), not only during the reproductive years, but also with aging and menopause; therefore, the early prevention and treatment of future cardiovascular adverse effects are necessary. The fundamental characteristic of PCOS is hyperandrogenemia, which is associated with increased pro-inflammatory cytokines and T lymphocytes. Whether these factors play a role in the pathophysiology of hypertension, a risk factor of CVD, due to PCOS is not well established. This review will briefly discuss how a modest increase in androgens in females is linked to the development of hypertension through pro-inflammatory cytokines and T lymphocyte subsets and the promotion of renal injury. Moreover, it reveals a few existing research gaps in this area, including the lack of specific therapy directed at androgen-induced inflammation and immune activation, thus emphasizing the necessity to explore the systemic inflammation in women with PCOS to halt the inevitable inflammatory process targeting the underlying abnormalities of CVD.

## 1. Introduction

**Figure 1:**
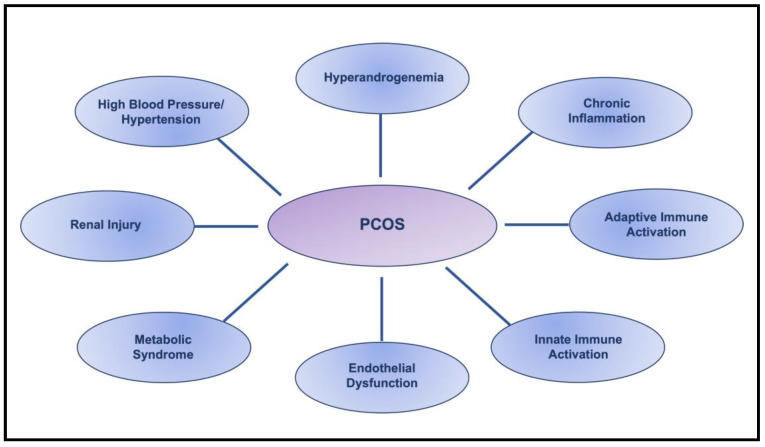
Summary of Cardiovascular Risk Factors in PCOS.

Polycystic ovary syndrome (PCOS) is the most common endocrine pathology in women of reproductive age, affecting more than 15–20% of the population, often beginning in adolescence [1,2,3,4,5,6]. The fundamental diagnostic symptom of PCOS is modest hyperandrogenemia [1,5,6]. In addition, PCOS is associated with substantial metabolic and cardiovascular long-term consequences. In young women with PCOS, several cardiovascular risk factors, including chronic inflammation, metabolic syndrome, hypertension, and cardiovascular events, may be found, even at an early age [4,7,8,9,10,11,12]. PCOS is strongly linked to cardiovascular diseases (CVD), the most significant and frequent cause of morbidity and mortality in women; whether this is true in women with PCOS across age and ethnic/racial groups is debatable. On one hand, many clinical studies report that women who have PCOS are at increased risk of CVD, not only during the reproductive years, but also with aging and menopause [13,14]. On the other hand, systemic reviews of population-based studies suggest that aging women with PCOS are not at a higher risk of CVD compared to healthy controls due to the decreased circulating androgens that occur as a consequence of aging [15,16,17,18]. Thus, the prevalence of CVD in women with PCOS across the age spectrum requires more investigation, especially as the treatment options for women with PCOS are limited and mainly focus on reducing insulin resistance and infertility. Furthermore, other variables, such as ethnicity and race, must be taken into consideration in future investigations because the prevalence of high blood pressure and hypertension is increased in Black women with PCOS compared to Caucasian, Asian, and Hispanic women [19]. Regardless of these controversial reports, the early detection of cardiovascular risk factors in women with PCOS is crucial for the prevention and management of CVD. Compelling studies have demonstrated that almost all cardiovascular risk factors, including chronic inflammation, metabolic syndrome, immune system alteration, endothelial dysfunction, kidney injury, and hypertension, are associated with PCOS [9,10,13,14,16,20,21,22], as illustrated in Figure 1.

Hypertension is recognized as one of the leading risk factors for CVD and approximately 30% of women with PCOS are hypertensive, compared to the general population of women aged 20–34 years [4,21,22,23]. While accumulating independent studies are being undertaken to explore the mechanism(s) of hypertension in PCOS, the exact mechanism(s) has not been elucidated. The evidence illustrates that the etiology of hypertension in PCOS is multifactorial; thus, several potential mechanisms contribute to the development of hypertension in PCOS, which require specialized attention and investigation. Chen and colleagues have demonstrated that hyperandrogenemia is associated with high blood pressure in women, regardless of obesity and insulin resistance [24]. Conversely, other studies have mentioned different factors, including obesity or insulin resistance, as risk factors for hypertension [25,26]. Recently, emerging evidence has called attention to immune cells and their cytokines, suggesting that hormones and immune cells orchestrate the chronic inflammatory state in PCOS. Taken together, these compelling studies bring forth the significance of the mutual impact of hyperandrogenemia, the inflammatory state, and the immune response, which may trigger endothelial cell dysfunction and renal injury, in mediating hypertension and ultimately, CVD.

So herein, we focus on highlighting a brief overview of our current understanding of how a modest increase in androgens in females may increase pro-inflammatory cytokines production, T lymphocytes activation, tissue infiltration of T lymphocyte subsets, kidney injury, and the elevation of blood pressure, and thereby the need for identifying possible unique therapeutic and lifestyle interventions that would be appropriate and specifically designed for women with PCOS. The lack of a specific therapy directed at the inflammatory mechanisms increases the necessity of investigating the chronic systemic inflammation and androgen-induced alterations in immune cells in women with PCOS to halt the inevitable inflammatory process.

## 2. Pro-Inflammatory Cytokines

While acute inflammation is a normal response of a healthy immune system to pathogens, with the purpose of healing the affected tissue and organ, prolonged inflammation has been linked to the development of chronic diseases, such as immune system dysfunction, hypertension, and CVD. Chronic inflammation is a common finding in women with PCOS, as measured by an increase in multiple markers of inflammation, such as C-reactive protein (CRP), tumor necrosis factor-alpha (TNF-α), interleukin-1β (IL-1β), interleukin-6 (IL-6), interleukin-17 (IL-17), interleukin-18 (IL-18), and interleukin-23 (IL-23), and a low level of anti-inflammatory interleukin-10 (IL-10) [27,28,29,30,31,32,33,34,35]. Therefore, PCOS is recognized as a pro-inflammatory state [9,36]. Several reports indicate that elevated levels of the pro-inflammatory cytokines TNF-α and IL-6 are well correlated with insulin resistance in women with PCOS [20], given that inflammation is also a common finding in individuals with increased visceral adiposity [37,38,39]. Despite these reports to the contrary, many studies have demonstrated that circulating inflammation is highly correlated with circulating androgen [9,36]. Additionally, a recent systemic review and meta-analysis showed that circulating inflammation is elevated in PCOS women, independent of obesity [40]. Similarly, it has been shown that developed animal models of PCOS exhibit significantly higher serum TNF-α and IL-6 concentrations compared with controls [41,42,43]. Many immune cells, including T lymphocytes, B lymphocytes, and macrophages, produce IL-6 and are activated in response to IL-6, resulting in autocrine feedback, which intensifies inflammation [44]. Accordingly, the up-regulation of pro-inflammatory cytokines is linked to the activation of various pathophysiological programs, such as pro-inflammatory, pro-atherosclerotic alterations, matrix remodeling, and apoptosis, that trigger vascular aging and impairment, which lead to CVD [45]. Therefore, chronic inflammation is strongly recognized as a predictor of coronary heart disease and CVD [45].

## 3. Immune Cells 

It is well established that chronic inflammation is also associated with increased leukocytes. There is growing research interest in leukocytes’ contribution to the inflammatory status in PCOS. Elevated circulatory pro-inflammatory T lymphocytes in women with PCOS, an increased number of T lymphocytes, B lymphocytes, and macrophages in the follicular fluid, and altered cytokine responses indicate the activation of the immune system in PCOS women, which leads to immune dysfunction [21,27,28,29,32,33,35,46,47,48,49,50,51,52]. While insulin resistance and obesity, not hyperandrogenemia, have been considered to be the main factors responsible for leukocytes elevation among PCOS women, by contrast, other confirmed reports have recognized androgen as an indicator of an increased leukocyte count in PCOS [31,32,48,50]. In addition, Rudnicka and colleagues have reported that the elevated leukocytes in women with PCOS compared to healthy subjects is positively correlated with androgen and insulin resistance [31]. Unfortunately, the exact mechanism of leukocytosis in PCOS has not been fully elucidated. However, collectively, there are compelling data to suggest that hyperandrogenemia is one of the main contributing factors in the development of leukocytosis and inflammation in PCOS. The identification of androgen receptors on the lymphoid and non-lymphoid cells of various immune organs, i.e., thymus and bone marrow [53,54,55], strongly suggest that hyperandrogenism, individually or in combination with insulin resistance, is responsible for elevated leukocytes [56]. Additional substantiation has been provided by an investigation of the therapeutic and cytotoxic effects of androgens against human leukemia cell lines in vivo and in vitro [57], as well as the direct effect of androgen on regulatory T (Treg) cells in women and its modulatory role in T lymphocyte differentiation [58]. The study has exhibited that, in women, Treg cells differentiate and proliferate in response to androgen exposure, and they are more responsive to androgen compared to their male counterpart. Taken together, these confirming studies indicate that excess androgen plays an important role in the activation and proliferation of leukocytes, particularly T lymphocytes, as they are sensitive to androgens during development and beyond, in contrast to B lymphocytes, which are mainly sensitive through development [53,54,55]. It is necessary to investigate the specific molecular mechanism(s) to fully understand the signaling pathway(s) in order to provide opportunities to develop novel therapeutic interventions for PCOS and the associated comorbidities. Remarkably, these studies may also serve patients afflicted by a variety of immune dysregulations; for example, understanding the proliferative effect of androgen on the Treg cell population in females could provide a new therapeutic intervention for autoimmune diseases.

## 4. T Lymphocyte Populations

It is well known that different cytokine environment profoundly affect the initiation of the adaptive immune response by differentiating naïve CD4 T lymphocytes into T helper (Th1, Th2, Th17) and Treg subsets upon T cell receptor (TCR) activation. A population of CD4^+^ T cells known as Th17 cells are characterized by their production of IL-17, which regulates tissue inflammation [59]. Pro-inflammatory IL-6 reduces the expression of X-linked forkhead/winged helix (FoxP3), but promotes the RAR-related orphan receptor-γ (RORγ) expression in naïve CD4^+^ T cells to promote Th17 differentiation into effector cells specialized in cytokine secretion and that have a distinct function [33,60,61,62,63]. Th17 cells, as pro-inflammatory T lymphocytes, promote the occurrence of inflammation by producing IL-17 and IL-23 cytokines; as a result of the broad distribution of the IL-17 and IL-23 receptors, a massive tissue reaction is induced. Th17 cells and their effector cytokines are being increasingly recognized as important mediators in inflammatory diseases [33,60,61,62,63]. Whereas transforming growth factor beta (TGF-β) induces Treg differentiation; the anti-inflammatory cells that inhibit inflammation by secreting anti-inflammatory cytokine IL-10 [64]. An imbalance of the Th17/Treg cells ratio occurs in inflammatory and metabolic diseases [33,65,66], suggesting that the balance between these two populations is vital in maintaining the homeostasis of the immune system.

Emerging studies have reported that CD4^+^ T lymphocytes are increased in PCOS patients [67,68]. Similarly, it has been revealed that the number of circulating CD4^+^ T lymphocytes is increased in hyperandrogenemic female (HAF) rats, an animal model that mimics PCOS [42]. Moreover, the immunological niche of blood, spleen, and kidney tissues in the hyperandrogenemia state are altered, which implies a potential immune system dysregulation in HAF rats compared to the placebo controls [42]. These findings may suggest a link between excess androgen, chronic systemic inflammation, and immune dysregulation in women with PCOS [42]. Moreover, substantial evidence has indicated that, in addition to the circulatory pro-inflammatory Th17 cell subset being elevated in PCOS women and animal models of PCOS, the anti-inflammatory Treg cell subset is significantly decreased [21,33,35,42,46,49,50,69]. Remarkably, the imbalance of Th17/Treg cells has been reported to be a critical factor in the pathogenesis of PCOS [35]. Additionally, the imbalance of pro- and anti-inflammatory T lymphocytes and their corresponding cytokines amplifies the inflammatory response that synergistically exacerbates chronic inflammation, mediating the cardiovascular adverse effect [33,70,71,72].

Despite the critical role of the Treg cell subset as an anti-inflammatory mediator in inflammation regulation, reproductive function, and preventing autoimmunity, its significance in PCOS remains largely uncharacterized; in addition, the frequency of the Treg population in hyperandrogenemia and PCOS is also controversial [73,74]. For example, Walecki and colleagues have demonstrated that, in women, Treg cells differentiate and proliferate in response to androgen treatment [58]. Conversely, growing studies have reported a significant reduction in circulatory Treg cells in women with PCOS, while another study has shown no significant changes in these cells [33,35,49,69]. Analogous to the situation in humans, several studies in animal models have indicated that female rats’ circulatory Treg cells expanded after androgen exposure [24,42,75]. Despite their expansion in the blood, Treg cells were significantly reduced in the spleen and kidney of HAF rats [42], suggesting a tissue-specific distribution in the hyperandrogenemic state and PCOS [42]. These findings suggest that androgen and mediated inflammation play an important role in either the up- or down-regulation of the Treg cell population; however, the specific mechanism(s) remains to be investigated to gain more insight into the role of androgen in the alteration of this T cell subset in women with PCOS.

Growing research has concentrated on the expanded circulating cytotoxic CD4^+^CD28^null^ T cells in PCOS to elucidate the PCOS pathophysiology, as their expansion is a consistent feature of the chronic inflammatory state [33,35,42,47,51,67]. Cytotoxic T lymphocytes eliminate infected or malignant cells by inducing cytotoxic activities. They secrete high levels of IFN*γ*, TNF*α*, and IL-2 cytokines and release cytotoxic molecules that amplify the inflammatory pathways [76,77]. Almost all normal CD4^+^ T cells express CD28 on their cell surfaces [78]. CD4^+^CD28^null^ cytotoxic T cells are rarely (<2%) found in healthy individuals, but are slightly expanded in the elderly, probably because of immuno-senescence; thus, a significant proportion of circulating CD4^+^CD28^null^ T cells is distinctly abnormal [79,80,81,82]. CD4^+^CD28^null^ T cell expansion is regulated by a complex cytokine network, particularly the exposure to TNF-α, which down-regulates CD28 expression and favors the expansion of this T cell population [77]. In all reported cases, CD28 down-regulation on significant proportions of peripheral CD4^+^ T lymphocytes is a specific indicator of an ongoing chronic inflammatory response, persistent inflammation-provoking infections, or other long-standing immunologic disorders [80,81,82,83,84,85,86,87]. Another important aspect is that they release cytotoxic granules containing perforin, granzyme A, and granzyme B, which may explain the infiltration of cytotoxic granule-laden lymphocytes into the inflammatory site [80,88]. Thus, activating monocytes and macrophages, alone and synergistically, may exert cytotoxic effects on inflamed tissues and damage endothelial cells [84,89,90,91,92]. Unfortunately, Treg cells are not capable of suppressing CD4^+^CD28^null^ T cells’ activity because they are resistant to the regulatory activity of Treg cells; hence, when they escape from Treg cells, they supercharge the inflammatory status [93]. Nevertheless, the triggers of CD4^+^CD28^null^ T cell activation have not been fully revealed and it remains controversial.

The significance of the CD4^+^CD28^null^ T cell population in inflammation, vascular, and renal diseases has been appreciated for decades; however, their contribution in the pathogenesis of PCOS and the associated comorbidities is under-explored. The limited studies indicate a significant increase in the CD4^+^CD28^null^ T lymphocyte population in women with PCOS and HAF rats that mimic many of the characteristics of PCOS; additionally, the association of perforin/granzyme-B levels with hyperandrogenism in PCOS strongly suggests a detrimental role in vascular diseases in PCOS [35,42,47,67,94]. Unfortunately, there has been very limited research conducted on the CD4^+^CD28^null^ T lymphocyte subset in animal models of hyperandrogenemia and PCOS. To the best of my knowledge, only one study has investigated CD4^+^CD28^null^ T lymphocyte subset alterations in the peripheral blood, kidney, and spleen tissue of a PCOS animal model [42]. Intensive research needs to be performed in different animal models of PCOS to address this gap in the knowledge. Moreover, it remains to be investigated whether the cytotoxic subset is activated as part of the systemic pro-inflammatory state in hyperandrogenemia and PCOS or due to an antigen-specific activation. Furthermore, whether T lymphocyte subsets activation is tissue-specific calls for further exploration. 

## 5. Hypertension

Hypertension, as one of the main cardiovascular risk factors, is significantly common among women with PCOS compared to controls [4,12,21,22,23]. However, despite years of research and confirmed reports supporting an association between PCOS and hypertension, the exact mechanism(s) has not been fully revealed. Compelling evidence suggests that several potential mechanisms contribute to the pathogenesis of hypertension in PCOS, which indicates that the etiology of hypertension in PCOS is multifactorial. 

Although the impact of hyperandrogenemia on the immune system in PCOS and animal models has generated attention in recent years as a potential cardiovascular risk in women, it is not well established, and the precise molecular mechanisms by which androgen exert an effect are not well elucidated. Emerging evidence firmly suggests that hormones and immune cells orchestrate the chronic inflammatory state and, consequently, may play a significant role in the pathogenesis of hypertension in PCOS. Considering that various studies have decisively established a role of the immune response and T lymphocytes in the pathogenesis of hypertension and in the tissue injury of hypertensive organs, the role of the combined effects of androgen-induced inflammation, immune activation, T lymphocytes infiltration, and an imbalance of Th17/Treg cells—which induce vasculature impairment and renal injury, thus mediating hypertension and ultimately CVD—require exploration as a potential mechanism involved in the pathophysiology of hypertension in hyperandrogenemia and PCOS.

The involvement of the immune system in hypertension has been well demonstrated, and it is becoming more evident that immune cells can affect blood pressure regulation [95,96,97,98,99]. Interestingly, it is suggested that the interaction between the innate and the adaptive immune responses is required for the development of hypertension. Innate immune cells, such as macrophages, microglia, monocytes, and dendritic cells, recognize new antigens that are released during oxidative stress, which leads to the activation of adaptive immune cells, including B and T lymphocytes [100,101]. Innate immune cells also produce cytokines and reactive oxygen species (ROS), which contribute to hypertension. Additionally, several studies have indicated that TNF-α and IL-6 play significant roles in the development of hypertension in different disease animal models [97,102]. TNF-α triggers the expression of vascular endothelial cells, and leukocyte adhesion molecules facilitate lymphocyte infiltration. TNF-α alone, similarly to IL-6, promotes the proliferation of effector T cells, and in combination with IL-6, provides highly effective synergistic helper signals for T cells proliferation [103,104,105]. Moreover, IL-6, as a critical co-factor, induces naive CD4^+^ T cells to promote Th17 differentiation and the secretion of IL-17 [33,63]. The up-regulation of inflammatory cytokines and the immune response, mediated by T lymphocytes, may directly lead to vascular impairment and an increase in blood pressure [99], as it has been shown that a modest elevation of leukocytes is associated with an increased risk of CVD and it has been identified as a predictor of coronary heart disease mortality, independent of the traditional risk factors [84,85,106,107,108]. 

An increased number of T lymphocytes in the follicular fluid, altered cytokine levels, and the expansion of pro-inflammatory T lymphocytes in PCOS women and animal models support a correlation between endocrine and immune system activation [27,33,35,42,47,52,109]. Additionally, accumulating evidence has confirmed the involvement of immune activation in hypertension and it is becoming increasingly evident that immune cells, especially the subtypes of T lymphocytes, can affect blood pressure regulation [95,96,97,98,99]. However, the exact mechanism by which the immune system plays a role in the pathophysiology of hypertension in PCOS is not well defined; therefore, the identity of the specific immune cells in androgen-induced hypertension in PCOS requires more investigation [110].

Given the essential role of the kidney in hypertension and the existing data on several animal models of experimental hypertension that are characterized by the infiltration of renal immune cells and the reduction in their infiltration and blood pressure after immunosuppressive drug administration [111,112,113,114,115,116], attention should be drawn to the renal-infiltrated T lymphocyte subsets as a mechanism contributing to the pathogenesis of hypertension in PCOS. It is well established that T lymphocytes play a crucial role in orchestrating the adaptive immune responses, infiltrating the kidney in hypertension, producing a large amount of pro-inflammatory cytokines, exerting cytolytic activity, and resisting pro-apoptotic signals [86,87,97,113,117,118]. Inflammatory cells recruitment from the circulation into the renal tissue is a typical feature of renal inflammation [119], which is facilitated by the chemokine receptors and adhesion molecules of these cells [101]. Infiltrating T lymphocytes mediate the initiation and progression of renal injury through the secretion of soluble mediators and/or direct cytotoxicity [120]. Collectively, these findings suggest that expanded T lymphocyte populations might be involved in the increased blood pressure, development or maintenance of hypertension, and long-term cardiovascular risk that PCOS women exhibit. Concurrently, inflammatory cell infiltration in the renal tissue, as well as an increase in inflammatory and cytotoxic secretion, can intensify chronic inflammation, damage the renal and vascular tissue and function, affect blood pressure, and mediate CVD. 

**Figure 2:**
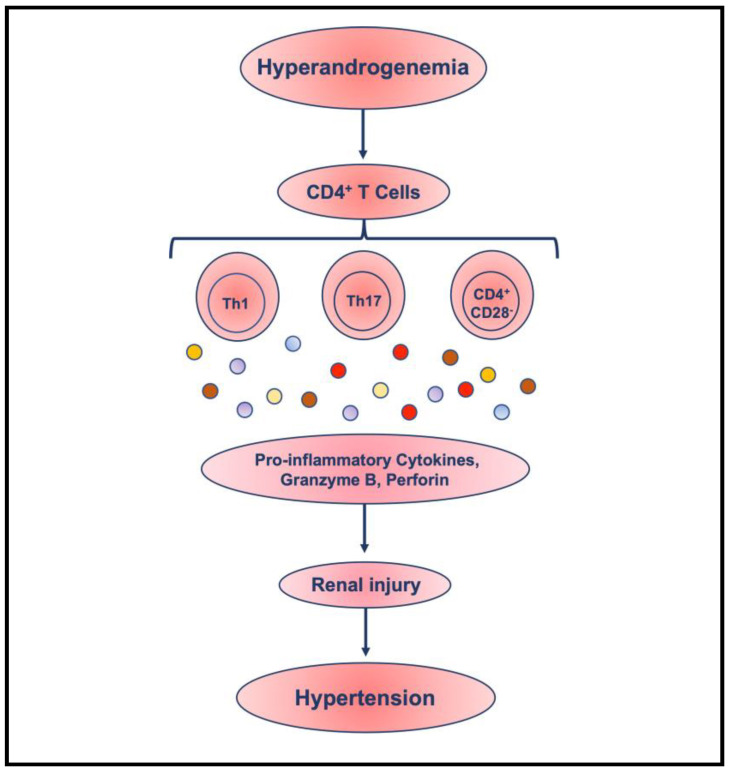
Schematic representation of the role of excess androgen in the pathophysiology of hypertension in PCOS, a synergistic relationship between pro-inflammatory cytokines and T lymphocytes mediating hypertension.

Furthermore, the expansion of the cytotoxic CD4^+^CD28^null^ T cell subpopulation in PCOS and the association of perforin/granzyme-B levels with hyperandrogenism in PCOS strongly suggest a high risk of immune-mediated CVD and kidney dysfunction in PCOS [94], similar to patients with acute coronary syndrome, atherosclerosis, renal diseases, and CVD [35,42,47,67,84,85,94,106,121,122]. Their increased frequency is considered a potential trigger for chronic vascular and cardiac inflammation, such as hypertension in women with PCOS, because it has been shown that they damage endothelial cells and the vascular tissue in both autoimmune disorders and coronary artery disease [35,47,106,123,124]. Importantly, an aggressive population of T lymphocytes has been associated with the recurrence of acute coronary instability, ischemic stroke, atherosclerotic, and CVD [85,121]. It is suggested that because they are capable of infiltrating into tissue to induce tissue injury, the presence of CD4^+^CD28^null^ cells at the site of inflammation will produce inflammatory cytokines, along with high levels of perforin and granzyme B, which may cause tissue damage to the endothelial cells. This can potentially result in an increase in atherosclerotic and initiate renal tissue injury, eventually leading to the progressive loss of renal function, contributing further to hypertension and CVD [93,120,122]. Therefore, further studies are required in both animal models and patients to investigate the potential mechanism involved in the pathophysiology of hypertension in hyperandrogenemia and PCOS of a synergistic relationship between pro-inflammatory cytokines and infiltrated T lymphocytes inducing renal injury and mediating hypertension (Figure 2).

While it has been demonstrated, in an animal model of hyperandrogenemia, that T cells depletion reduces blood pressure [125], it remains to be investigated whether the engaged T lymphocyte populations are activated as part of the systemic inflammatory state in PCOS or, rather, that an antigen-specific activation of different T lymphocyte populations might be involved. Furthermore, it should be noted that pro-inflammatory cytokines are not secreted exclusively by T cells, which represents an important caveat in this research field. Additionally, the role of B cells as a key player in the adaptive immune response in CVD in PCOS remains obscure. B cells multitask by presenting processed antigens for T cell activation, producing pro- and anti-inflammatory cytokines, and secreting antibodies. Remarkably, convincing evidence has been provided for the contribution of B cells in hypertension in recent years [126,127,128]. Therefore, the role of B cells in hypertension and their response to excess androgen in PCOS remain to be explored. 

## 6. Pregnancy-Induced Hypertension

Women with PCOS are at a significantly increased risk of pregnancy-related complications compared to controls [129]. Several meta-analyses have reported a significantly increased risk of hypertension, of three to four times, in women with PCOS during pregnancy [130,131,132,133,134,135]. These studies highlight the increased risk of maternal CVD associated with pregnancy-induced hypertension (PIH) in women with PCOS, regardless of the existing risk factors before pregnancy [136,137,138]. In contrast, a new study by Yang and colleagues found that there is no increase in the risk of PIH among Korean women with PCOS [139]. Therefore, the significant heterogeneity in the samples included in the meta-analyses calls for more investigations based on population ethnicity and diagnostic criteria, especially due to the extensive ethnicity variations in the manifestation of PCOS. 

The maternal immune response to pregnancy is trackable through changes in the circulating cytokine profiles. The normal immune response to pregnancy is indicated by the development of immunological tolerance to paternally derived antigens expressed by the fetus, which is characterized by an increase in the levels of IL-10 positive cells, a decrease in the T helper 1/T helper 2 (Th1/Th2) ratio with a shift to Th2 dominance, and, consequently, cytokine profiles alteration [140]. The failure to develop immunological tolerance is defined by a decrease in IL-10 positive cells, an increase in the levels of IL-17 positive cells, and a failure in a shift to Th2 dominance [141,142]. These changes can result in a serious pregnancy complication. Pregnancy in women with PCOS is associated with higher levels of circulating inflammatory molecules, such as CRP, IL-6, TNF-α, and IL-17 cytokines [143,144,145], and increased leukocytes in the peripheral blood [143] throughout pregnancy compared to controls, which indicate a more activated immune system compared to controls. Moreover, it has been shown that inflammation induces leukocyte–endothelium interactions and increased levels of adhesion molecules, which may be aggravated by the presence of insulin resistance [144] and androgen-mediated chronic inflammation in PCOS. Elevated adhesion molecules facilitate the renal infiltration of pro-inflammatory cells, as mentioned earlier [101]. Ultimately, these studies suggest that pregnancy exacerbates the existing chronic inflammation in women with PCOS, as indicated by leukocytosis and increased inflammatory molecules [143,144,145], which leads to vascular impairment, an elevation in blood pressure, and an increased risk of CVD.

The limited available data about blood pressure levels in women with PCOS during pregnancy calls for more pre-clinical and clinical studies. Additionally, while some studies have indicated a higher risk of PIH in women with PCOS compared to controls [130,131,132,133,134,135], this has not been confirmed in women with PCOS of Asian ethnicity [139]. Most of the existing data have been collected from selected study cohorts of women with PCOS with a significant heterogeneity; therefore, further investigation is essential to clarify all of the previously mentioned experimental and clinical aspects of PIH and future maternal cardiovascular risks in PCOS from a diverse ethnicity background. Moreover, it remains to be established whether androgen-mediated inflammation is an individual risk factor for PIH or other PCOS-associated comorbidities. This will pave the way for the future interventional research aimed at preventing and/or reducing CVD in mothers with PCOS.

## 7. Autoimmune Thyroid Disease and Hypertension

There is a growing body of evidence demonstrating an association between PCOS and autoimmune diseases. PCOS has been recognized as an inflammatory state [9,36,42], defined by the up-regulation of pro-inflammatory cytokines, imbalance of T lymphocytes, and alteration of most immune system elements [32,33,42]. Chronic inflammation has been suggested to be a potential link connecting an increased prevalence in a variety of autoimmune conditions, especially autoimmune thyroid diseases (AITD) [146,147,148], which are also associated with inflammation and altered immune cells. While the recognizable connection, perhaps, is the immune and metabolic dysfunction common to both disorders, the pathophysiological pathway(s) linking these two disorders has not been well demonstrated. 

The effect of thyroid dysfunction on the cardiovascular system may increase the risk of hypertension [149,150,151], and it is considered to be a cardiovascular risk factor [151,152]. The high-risk correlation of thyroid dysfunction coinciding with PCOS amplifies the CVD risk among women with PCOS [153]. Therefore, these factors should be taken into consideration as markers of the future incidence of CVD in PCOS patients. Consequently, screening for thyroid-specific autoantibodies and thyroid function should be considered in these patients, regardless of thyroid dysfunction-related symptom manifestations. Additionally, a pragmatic approach is required for future investigation to identify an approach to prevent and/or treat thyroid dysfunction in patients with PCOS.

## 8. Basic Intervention

Lifestyle modification has been recognized by the Androgen Excess and PCOS Society and International PCOS network as a primary choice in the management of PCOS and the associated comorbidities, including androgen-mediated inflammatory status, endothelial dysfunction, high blood pressure, and CVD [4,7,10,154,155,156,157]. Women with PCOS are required to seek evidence-based educational opportunities to learn about foods with anti-inflammatory properties, commit to diet modification [158,159], and be involved in moderate physical activity at an early age to alleviate metabolic dysfunction and improve their blood pressure to reduce the risk of CVD. 

Indisputable documentation supports the health benefits of being physically active and it is well established that exercise improves the endothelial function and Th17/Treg imbalance, and thereby prevents cardiovascular events in hypertensive patients [160,161,162,163]. As the endothelium plays an important role in the pathogenesis of hypertension and CVD, its functional biomarkers are measured to assess cardiovascular risk. Remarkably, an improvement in the marker of endothelial dysfunction, endothelial cell-derived microparticles, has been documented after supervised, moderate-intensity exercise [164]. Furthermore, high circulating leukocytes in women with PCOS have been reversed by aerobic exercise [165]. Lower inflammatory biomarkers, IL-6, TNF-α, and CRP, have also been observed in women with PCOS, and lower IL-6 and TNF-α in an animal model of hyperandrogenemia and PCOS after aerobic exercise [166,167]. In contrast, Thomson and colleagues have reported that exercise provided no additional benefit on the markers of endothelial function in obese PCOS women with a high-protein, low-caloric diet [168]. Moreover, several studies have reported higher levels of pro-inflammatory cytokines after intense, long exercise, which promotes robust chronic inflammation [169]. This calls for further comprehensive investigation and assessment to address these controversial reports in hyperandrogenemia and PCOS patients. 

## 9. Discussion

In this review, a potential link between excess androgen, the inflammatory state, altered T lymphocyte subsets, renal injury, and hypertension as risk factors of CVD in animal models of PCOS and women with PCOS, including non-pregnant and pregnant women, were discussed. In summary, the existing studies confirm that the increased prevalence of hypertension in PCOS is considered a significant contributor to the elevated risk of CVD. Moreover, these studies suggest that alterations in the immune response may be a potential mechanism involved in the pathophysiology of hypertension in hyperandrogenemia and PCOS. These findings, combined with the accumulating evidence, indicate that a modest increase in androgens in females contributes to increased pro-inflammatory cytokines and infiltrated T lymphocyte subsets in the kidney, inducing renal injury, and thus mediating hypertension. Moreover, an elevation in blood pressure may also intensify existing endothelial damage and kidney injury, which in turn induces inflammation and exacerbates high blood pressure. 

One strength of the available studies is that the roles of androgen-induced immune system alteration and hypertension have been recognized, and evidence indicates that hyperandrogenism is an essential factor in the inflammatory status of PCOS, which affects multiple cells and tissues. However, there are several limitations: one limitation of the available data concerns the fact that the data have been collected from select cohorts of women with PCOS which indicate the lack of cohort heterogeneity. Therefore, a significant heterogeneity in the samples included in the meta-analyses calls for more investigations based on population ethnicity and diagnostic criteria, especially due to the extensive ethnicity variations in the manifestation of PCOS. It is essential for further studies to be conducted to include women with PCOS from different geographical regions and different ethnicities and races. Another concern is the limited data availability regarding PIH in animal models of PCOS; this paucity calls for more experimental and preclinical studies. 

Furthermore, in this review, a gap in the knowledge of the CD4^+^CD28^null^ T lymphocyte population in animal models of PCOS was identified. Similarly, a gap in the knowledge on the CD4^+^CD28^null^ T lymphocyte population in pregnant women with PCOS was also recognized. Accordingly, it is recommended to explore the role and mechanism(s) of the CD4^+^CD28^null^ T cell population in the pathogenesis of PCOS and the associated comorbidities, including hypertension, PIH, AITD, and CVD, thereby providing a better understanding of androgen-induced inflammation and the immune alteration process. Finally, there is a significant gap in the knowledge regarding the screening, evaluation, diagnosis, and treatment of PCOS patients with AITD comorbidity. While, based on earlier studies, the association between autoimmune thyroid disease and PCOS is being increasingly recognized and it is well established that thyroid dysfunctions have negative impact on the cardiovascular system, and especially hypertension, which may create an amplification event in PCOS patients however, the concurrence of these conditions is still insufficiently explored.

From further experimental research, one may learn additional preventative measures, such as markers for screening to be examined in clinical practice. Unfortunately, the impact of excess androgen on the immune system in females and women with PCOS is not well established. In particular, the effect of high levels of androgens in females and its role in immune dysfunction and expanded lymphocytes as essential participants in the development of hypertension and PIH in PCOS requires additional exploration. Further investigations demonstrate that not only, increased pro-inflammatory cytokines, but also altered T lymphocyte subsets, can be used as potential targets for therapeutic intervention to prevent and/or reverse hypertension as one of the main risk factors of CVD in pregnant and non-pregnant women with PCOS. Furthermore, experimental and clinical investigations on thyroid dysfunctions and associated negative impact on the cardiovascular system in patients with PCOS may lead to routine screening for thyroid dysfunction in clinical practice, earlier management, and regular follow-up to offer a greater assurance of CVD is necessary.

Finally, the controversial evidence regarding the impact of physical activity in women with PCOS requires further in-depth investigation. Several questions remain to be answered by the experts in the field, including: does exercise divert the balance between Th17 and Treg cell subsets in hyperandrogenemia and/or PCOS?; what is the ideal exercise regimen for women with hyperandrogenemia and/or PCOS?; what is the optimal exercise routine for young vs. postmenopausal women with PCOS?; and what is the optimal exercise routine for women with PCOS during pregnancy? 

## 10. Conclusions

In conclusion, the existing controversy about the exact role of excess androgen in hypertension in PCOS signifies a clear future research goal(s) and the necessity to develop strategies for the timely screening and diagnosis of hypertension in PCOS patients throughout the lifespan. Nevertheless, to understand the immune etiology and pathogenesis of PCOS, associated hypertension, and CVD, further investigation into the immunological mechanisms and pathways is required, which may lead to immunological intervention and the development of novel therapeutic prevention and treatments for androgen-induced immune alterations and inflammation. This would ultimately lead to tailored strategies to screen, diagnose, and risk-stratify women with PCOS and associated coexisting conditions to prevent and/or treat the associated comorbidities, including hypertension and CVD. 

## Data Availability

Not applicable.
